# Healthcare provider’s perspectives on home blood pressure management in Peru and Cameroon: Findings from the BPMONITOR study

**DOI:** 10.1016/j.pmedr.2023.102179

**Published:** 2023-03-17

**Authors:** Tala Al-Rousan, Mina Awad, M. Amalia Pesantes, Namratha R. Kandula, Mark D. Huffman, J. Jaime Miranda, Rafael Vidal-Perez, Anastase Dzudie, Cheryl A.M. Anderson, BP MONITOR Study Group

**Affiliations:** a Department of Medicine, University of California San Diego School of Medicine, United States; b Herbert Wertheim School of Public Health and Human Longevity Science, University of California San Diego, United States; c Universidad Peruana Cayetano Heredia, Lima, Peru; d Department of Anthropology and Archaeology, Dickinson College, United States; e Departments of Medicine and Preventive Medicine, Northwestern University Feinberg School of Medicine, United States; f The George Institute for Global Health, University of New South Wales, Australia; g CRONICAS Centre of Excellence in Chronic Diseases, Universidad Peruana Cayetano Heredia, Lima, Peru; h Department of Cardiology, Complejo Hospitalario Universitario de A Coruña, A Coruña, Spain; i Clinical Research Education, Networking and Consultancy (CRENC), Yaounde, Cameroon

**Keywords:** Hypertension, Blood pressure, Self-management, Medication, Qualitative, Peru, Cameroon, Healthcare providers

## Abstract

Home blood pressure management, including self-monitoring and medication self-titration, is an efficient and cost-effective tool. Although its use is increasing globally, little is known about the feasibility of such interventions in low and middle-income countries. Further, the perspectives and experiences of healthcare providers who play a big role in ensuring the success of home blood pressure management interventions have not been documented. This qualitative study was conducted with a total of 35 healthcare providers (60% female, mean [SD] age = 37.3 [6.9 years] years), through 4 in-depth interviews from Peru, and 8 in-depth interviews and 4 focus groups from Cameroon. Study participants (healthcare providers) include physicians (primary care physicians), specialists (cardiologists and geriatricians), and nurses that were purposively recruited from two hospitals in two of the largest cities in both countries. Results were thematically analyzed by two researchers. Themes derived were related to feasibility and acceptability, and largely reflected providers in both countries endorsing home blood pressure management. Providers’ concerns were in three main areas; 1) safety of patients when they self-titrate medications, 2) resources such as healthcare financing, local hospital policies that support communications with patients for home blood pressure management, and 3) sustainability through patient adherence, incorporating home blood pressure management within clinical guidelines and hospital policies, and complementing with continued health education and lifestyle modifications. According to providers, home blood pressure management may be feasible and acceptable if tailored multi-faceted protocols were introduced bearing in mind local contexts.

## Highlights

• Home BP management may be feasible in Peru and Cameroon if tailored to local context.
• Safety, resources, and sustainability were main concerns for healthcare providers.
• Adherence, accessibility, and affordability of med. and BP monitors were challenges.
• Physician-patient relationship is key in adherence; self-monitoring can add burden.
• Systems needed for longevity & med. self-titration of home BP management programs.

## 1 Introduction

Hypertension is a leading global public health challenge and accounts for 13% of total deaths globally. The number of people aged 30–79 years with hypertension doubled from 1990 to 2019, with many low- and middle-income countries (LMICs) reporting the highest rates of uncontrolled hypertension (Worldwide trends in hypertension, 1990). Self-management of hypertension including self-titration of medications has proven to be a cost-effective intervention that achieves better blood pressure (BP) control in various parts of the world (McManus et al., 2018, Bosworth and Crowley, 2015).

Self-management is also one of the 6 essential elements identified in the Chronic Care Model for chronic disease management (Wagner, 1998). The Chronic Care Model seeks to improve quality and outcomes by focusing on system-level changes that have an impact on patient- and provider-related factors (Wagner et al., 2001). This includes buy-in from healthcare providers who play a major role in the support of BP self-management interventions. Healthcare providers including nurses can influence health behaviors and engagement that affect the outcomes of BP management. Using self-management support in primary or specialty care can have a positive effect on the care and health outcomes of people with chronic conditions, as well as provider and patient satisfaction (Abdullah et al., 2016, McManus et al., 2018). However, the majority of literature on the experiences, barriers, and facilitators to hypertension self-management and BP management from the perspective of healthcare providers comes from high-income countries only (Khatib et al., 2014). Offering patient-centered BP treatment will require patient and, healthcare provider collaboration, yet there is a paucity of knowledge about the feasibility and acceptability of home BP management from the healthcare provider perspective, particularly in LMICs settings. A study of experts from Asia concluded that home BP management is low in Asia but can be a promising intervention (Wang et al., 2021).

The BPMONITOR study was designed to examine barriers and facilitators to self-management of hypertension including home BP monitoring and medication self-titration (Anderson et al., 2017). Barriers and facilitators from patients’ perspectives from this study have been published elsewhere (Al-Rousan et al., 2020). Herein, we present data from interviews conducted with healthcare providers that care for patients with hypertension from Cameroon and Peru. Together, the findings are instrumental in describing the feasibility and acceptability of contextualizing and potentially adapting future BP self-management interventions to achieve optimal BP control in LMICs.

## 2 Methods

### 2.1 Design

The BPMONITOR study was a multi-site pilot developed to examine the feasibility of home BP monitoring, development, and execution of a stepped-care self-titration medication plan with healthcare providers in multiple countries according to standard of care. These LMICs included Peru, Cameroon, and Malawi. However, data from healthcare providers were only successfully collected from only Peru and Cameroon due to budgetary constraints. The goals, protocol, and theoretical framework of BPMONITOR and study guides used have relied on the Consolidated Framework for Implementation Research (CFIR) framework and have been previously reported elsewhere (Anderson et al., 2017). In the current study, we conducted individual in-depth interviews in Peru and Cameroon and focus group discussions (FGD) in Cameroon with healthcare providers from different medical specialties who routinely care for patients with hypertension. *We utilized grounded theory for this study to develop data-driven theories on patient centered blood pressure management.* (Chapman et al., 2015) *The generation of theory and formulated conclusions were based on collection and analysis of data.* Our reporting adheres to the Criteria for Reporting Qualitative research (COREQ) guidelines. Ethical compliance approval for this study was granted by the Universidad Peruana Cayetano Heredia’s Institutional Review Board (CIE: 66238) in order to conduct human subjects research within Peru; the Hospital Santa Rosa Hospital approval was obtained to allow research staff to approach healthcare providers for interviews within the hospital in Peru, and the Deido District Hospital Research Ethics Committees ethical approval was obtained for human subject’s research within Cameroon.

### 2.2 Participants and setting

Qualitative data were collected between June 2016 and May 2018 in Lima, Peru, and from March 2016 to May 2019 in Douala, Cameroon, using a standardized protocol and maximum variation sampling, through convenience sampling in Cameroon and purposive recruitment in Peru, by non-randomly sampling both physicians (e.g., primary care physicians) and non-physician (e.g., nurses) as well as sampling from different specialties (e.g., cardiologists and geriatricians). The term ‘health care providers’ includes physicians (i.e., primary care physicians, and specialists such as cardiologists and geriatricians), and nurses. In Peru, specialists were recruited from cardiology and geriatric outpatient departments affiliated with Hospital Santa Rosa, a public tertiary hospital to ensure diversity of healthcare providers. This hospital is managed by the Ministry of Health, is located in the city center of Lima, and serves 5 districts of Lima. In Cameroon, participants were recruited from the cardiology department of Deido District hospital, a hospital in the center of Douala, which is the economic capital of Cameroon. The hospital serves the entire Deido district and 4 neighboring areas (i.e., Akwa, Bonamoussadi, Makepe, and Bonaberi), which includes a mix of middle- and low-income Cameroonian patients. Healthcare providers were approached by the study team and were asked for their availability to schedule their interviews. We organized such that we would not interfere with workflow or clinical care and this approach helped to ensure that research participation.

Healthcare providers were recruited to participate in the study at both sites if they cared for patients with a diagnosis of essential hypertension regularly. A purposive sample of healthcare providers to obtain equal numbers of males and females were sought. Trained research assistants approached providers in clinics making sure not to interrupt clinical workflow. Healthcare providers who agreed to participate were consented and agreed on a time and location for the interview. Those who have not agreed to participate were recorded as “declined”. Age group, type of healthcare provider (e.g., physician, specialist, nurse, and community health worker), specialty, and practice location were also purposefully taken into account during recruitment in Cameroon to ensure generalizability. In Peru, local hospital ethical approval only allowed for the recruitment of physicians and specialists since nurses and community health workers needed to be compensated for their time beyond the funding available for the study. Interviews at each site lasted between 45 and 60 min and occurred in a separate office dedicated to conducting the study within the clinic.

### 2.3 Data collection

We designed and piloted a semi-structured guide (Table 1) for conducting the interviews and focus group discussions with healthcare providers. This guide was developed within an implementation science framework of context, outer, inner, processes, interventions, and individuals (Anderson et al., 2017). It was designed to explore the experiences and perspectives of healthcare providers (Al-Rousan et al., 2020) about patients’ diagnosis of high BP, the regularity and approach to measurement, the adherence and access to treatment, the patient-physician relationship, and healthcare providers’ opinions about monitoring their BP and self-titrating medications at home.

**Table 1:** A semi-structured interview guide, resulting themes and representative quotes about provider perspectives on self-management of blood pressure.

Theme	Representative Quotes
**Theme 1.** Experience and Attitudes towards High Blood pressure	
A1. How big a problem is high blood pressure in your patient population? Subthemes: • High patient volume • Misdiagnosis • Stigma • Health literacy • Comorbidities	1.a *“We see hundreds of patients per month who have hypertension. From those, I would say 70% have really high readings when we measure their blood pressure in the clinic despite being on medications.” (Nurse, Cameroon)*
	*“The patient comes with a referral notice that says one thing but when I examine the patient they have nothing. The primary clinics are not doing their jobs correctly in documenting data. They even send us children to rule out ischemic heart disease and essential hypertension. That makes no sense.” (Cardiologist, Peru)*
	“*The education level of the average Peruvian has improved in recent years, so now the majority of the people have at least finished high school. Yes, the majority of the people do understand what hypertension is, I mean, the first thing I tell them is that it is a chronic illness and that it cańt be cured and they have to understand that they will need to take medications for life or at least for many years to come, that is the concept I like to convey right from the start what they are dealing with. The higher your education the more likely they will believe me.” (Cardiologist from Peru)*
	*“Firstly, probably it is a problem of means to get to the hospital and take care of your health, and secondly, a little bit of ignorance. Patients come with complications and very high blood pressure readings.” (Primary care physician, Cameroon)*
	*1.e “At a certain age when a patient is over 80 years you have to have a low-salt diet. When I tell this to a patient, they refuse to follow these orders stating that a pharmacist told them that with age their kidneys releases salt so they cańt lower the salt in their diet so much. This type of misinformation makes it more difficult to manage hypertensive patients.” (Geriatrician, Peru)*

**Theme 2.** Accurate Measurement of Blood pressure	
B1. Do you ever have patients monitor their blood pressure at home or outside the clinic? What do you ask them to do? Why do you ask them to do this? Subthemes: • Lack of access to monitors • Cost • Not measuring correctly (especially in the case of older age and co-morbidities) • Lack of guidelines (inconsistency in prescribing)	*“I ask patients to use ambulatory blood pressure monitoring (ABPM) but having them get an ABPM sometimes takes a month, two months and sometimes they cannot even get them at all. A way to evaluate this is by having themselves take their blood pressure, I tell them to go to the health center or to a pharmacy and write down their blood pressure readings.” (Cardiologist, Peru)*
	*“We have patients who can afford these electronic machines; they purchase it and measure their blood pressure at home, but they are only a handful. Those who can’t measure their blood pressure at home are the ones who can’t afford to pay for the machines because having the machine is the key factor and none covers the cost of them except the patients themselves.” (Primary care physician, Cameroon)*
	*2.c “Sometimes we ask them to bring the device to see if they are taking their blood pressure the right way, if it is working well because we have to show them if the cuff is for the wrist or for the arm, if they are reading properly the instructions, if it is calibrated because sometimes it is not calibrated and they say that that their blood pressure is normal and when we take the blood pressure here with the clinic’s blood pressure monitor it is very different.” (Geriatrician, Peru)*
	*2.d “Usually, patients dońt understand the handling instructions of the device. There are a lot of numbers, and they dońt know their meaning especially if they cannot see well.” (Geriatrician, Peru)*
	*2.e “The clinical guidelines keep changing but the hospital also has different guidelines for us” (Primary care physician, Cameroon)*

**Theme 3.** Treatment of High Blood pressure	
C1. Do you think most of your patients with high blood pressure follow the instructions/advice you give them? *Can probe more specifically*: about their medicine or lifestyle? Subthemes: • Medication adherence • Access to medications • Patient activation • Political influence • Difficulty changing other lifestyle behaviors (i.e.; diet, exercise)	*“Sometimes patients call me saying: “I cańt find valsartan, what can I take instead?” and that is in the best of the cases when they come to me and ask, there are other patients who dońt ask, they go to the pharmacy and buy a similar medication or any other medication for high blood pressure after asking the drug store worker without considering if there is a contraindication. Plus, the availability of drugs is a problem, because here in the pharmacy we don’t always have the drugs that we need.” (Cardiologist, Peru)*
	*“I prefer to give a single or two dose either in the morning and/or evening that are right after or around the meal times, because if I prescribe the medication on a fixed schedule they tend to stick to it and not forget.” (Geriatrician, Peru)*
	*“First and foremost, the government and policy makers can begin by subsidizing the cost of hypertensive work-up because the tests are many and expensive and many preventive tests are not covered by health insurance.” (Nurse, Cameroon)*
	*“Patients find it difficult to change their lifestyle such as diet, but it is our duty as health personnel to make them understand the importance of dietary modifications for blood pressure control before we talk about extra steps at home with a blood pressure device.” (Nurse, Cameroon)*
	*3.e “There are patients that tell you “No,”, and are difficult [patients]. They tell me: “I already suspended the medication because my blood pressure measurements were ok.” I explain: “You are a controlled hypertensive patient but that is because you are taking medications but if you suspend them, you will have high blood pressure again.” (Cardiologist, Peru)*
	*3.f “Cardiovascular diseases, although they are the second cause of death in the country, are not valued in their real dimension. The people here continue to put money into only infectious diseases and strategies to deal with them. The only political force lobbying for non-communicable diseases was the one lobbying for Cancer with the Plan Esperanza (Peruvian National Plan for Cancer Prevention and Treatment). We need something similar for cardiovascular diseases”. (Cardiologist, Peru)*

**Theme 4.** Patient-Physician Relationships	
D1. Tell me how you see your role as a physician in helping patients with high blood pressure? Subthemes: • Give patients agency • Cultural norms	***“*** *At the end of the day, my patients are adults, and I am not their parent, they must decide what works and what does not work well for them, I give them the information and if they decide not to take the pills they are the ones who will suffer the consequences.” (Cardiologist, Peru)*
	*“If a patient can follow his treatment, not smoke or take alcohol and comes for blood pressure control, it improves our relationship, and we can proceed to different treatment options.” (Primary care physician, Cameroon)*
	*4.c “I provide help like giving additional appointments to come to the hospital. I used to work at a private clinic, but I quitted because I always give my cellphone to be available 24 h a day and patients used to call me at midnight saying “ doctor, my pressure is high”, “ how much?”, “ I dońt know, I look very red”, I reply: “Maybe because it is summer and you have spent too much time in the sun.” (Cardiologist, Peru)*

**Theme 5.** Opinions about Self-management and Medication Self-titration	
E1. I am proposing to you a program where you ask patients to measure their blood pressure at home and based on their weekly average blood pressure, they can change their medications as you instruct them to do so in advance. Would you be interested in you and your patients participating in such a program/intervention? Why? How do you think this could benefit you? Is there anything that worries you? Subthemes: • Sense of agency for decision making/authority • Feasibility • Social support • Training	*“Self-management study might work out better with someone who had hypertension for a long time, and they know that blood pressure can fluctuate but for newly diagnosed patients they will just do whatever they want with the medications because they do not know or trust their physician.” (Cardiologist, Peru)*
	“*Practicing self-monitoring first of all eliminates the white coat effect so it is very important and is being practiced all over the world but here we need a push to start doing it.*” (Primary care physician, Cameroon)
	*“Someone has to be by these patients to guide them during their medication intake, someone who is able to purchase their meds when they get finished. Social support is key in everything about chronic disease management.” (Primary care physician, Cameroon)*
	*5.d “Let’s say we have a stable patient, that is already taking medications, to have him or her take his or her blood pressure and to change their medications I would be going against everything I said. If a patient has their blood pressure well under control and he or she reduces in half their doses and again starts having high blood pressure, there we can say we have a problem, and the doctor does not know what he is doing.” (Cardiologist, Peru)*
	*5.e “I think it will make the patient play an active role in their blood pressure management not just being passive and when a patient is able to monitor his blood pressure at home and knows when to adjust his medications, that patient grows and take better care of their overall health.” (Nurse, Cameroon)*
E2. What would make life better for those with high blood pressure? What would make it easier for you to take care of patients with high blood pressure? Subthemes: • Combining with lifestyle interventions/education programs • Multi-level support and policies	*5.f “Cardiovascular diseases, although they are the second cause of death in this country, are not valued in their real dimension. Government continues to put money into infectious diseases and prevention strategies. We need more resources channeled into cardiovascular health prevention” (Cardiologist, Peru)*
	*5.g “It is important to have a hypertension program at the hospital level and hire dedicated personnel to keep track of these patients, counsel them for lifestyle interventions or health promotion activities and administer besides the self-management programs” (Primary Care Physician, Cameroon)*

Upon invitation, healthcare provider interviews were conducted in the study office. The average (mean, SD) time used for in-depth interviews was 45 min (SD: 17 min) in Peru and 35 min (SD: 25 min) in Cameroon, respectively. The mean interview time for focus group discussions in Cameroon was 42 min (SD: 25 min). In Peru, interviews were conducted by a female researcher with a doctoral degree in medical anthropology, and in Cameroon, interviews were conducted by a male researcher who is a community health worker. Interviewers were trained to conduct qualitative research, and all interviews were administered in the local language, performed at the health facilities, and were audio recorded after obtaining written consent from all participants. Data collection continued until thematic saturation was reached where no new themes emerged (Guest et al., 2006).

### 2.4 Data analysis

Data from in-depth interviews were put into matrices and organized around six predefined topics from the interview/focus group guide (Table 1). We used inductive and deductive thematic analysis to identify key themes where multiple experiences and perspectives contributed to developing the final research outcome. Interview recordings were charted in the original language, then translated and verified by two others.

Relevant quotes for each topic were selected, and then two study team members per site organized the information for analysis and interpretation. The interview data were analyzed and transcribed through an iterative process using ATLAS.ti, a qualitative data analysis software. A team of 3–5 coders inductively coded data from 5 out of 16 transcripts, meeting regularly to review the codes as a team. We discussed and resolved discrepancies in coding and developed a codebook based on consensus among coders. The codebook was reviewed by the first, second, third, and senior authors. The first author (TA) then used this codebook to code the remaining transcripts. When new codes emerged, they were reviewed by the multidisciplinary team during regular meetings and added to the codebook. Any disagreements were discussed and resolved. We reached thematic saturation when no new codes emerged, though we continued to code all of the transcripts.

**Fig. 1:**
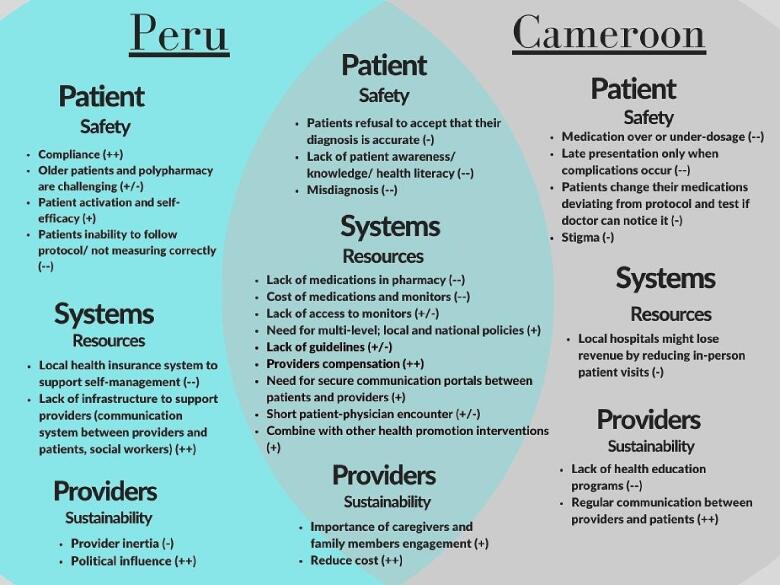
Overview of main influential factors on the implementation of home blood pressure self-management in Cameroon and Peru ordered by Consolidated Framework for Implementation Research domain and (sub-) constructs. Legend: + positive / ++ very positive influence; − negative / −− very negative influence; +/− mixed influence.

Our team identified themes and organized them into overarching domains based on the goals of the study: overall attitudes and BP measurement, treatment, patient-physician relationship, characteristics of local health systems, and perceptions about home BP management interventions that include BP self-monitoring and medication self-titration. We then deductively organized the sub-themes and constructs within the feasibility and acceptability domains into a structure based on the CFIR framework (Fig. 1).

## 3 Results

### 3.1 Participant characteristics

In Peru, we conducted 4 in-depth interviews, and in Cameroon we conducted 8 in-depth interviews and 4 focus group discussions. In Peru, 4 of 5 healthcare providers who were contacted agreed to participate and completed the study. In Cameroon, 8 of 10 healthcare providers who were approached for in-depth interviews agreed to participate, and 23 of 26 healthcare providers participated in focus group discussions.

In Peru, 2 cardiologists (1maleand1female) and 2 female geriatricians participated in the interviews. In Cameroon, 8 community health workers (4female), including 4 physicians (3primarycarephysiciansand1specialist) and 4 nurses participated in the in-depth interviews. Each focus group discussion had 5–6 participants all of whom were primary care physicians. The mean age was 37.3 ± 6.9 years (range: 22–42 years).

### 3.2 Theme 1 from Interviews: experience and attitudes towards high blood pressure

In both Peru and Cameroon, levels of health awareness and sources of information related to high BP were concerns of healthcare providers. This lack of awareness among patients included misconceptions about the disease. Healthcare providers stated that some patients argue that their diagnosis of essential hypertension is temporary and can be reversed. Healthcare providers reported that most patients think that one BP measurement is enough to take action on medications, expecting physicians to change medications at each clinic visit. In both countries, healthcare providers believed that uncontrolled BP is a big problem among their patient population. In Peru, cardiologists revealed that each healthcare provider cared for 195–210 patients per month in 2019, and 30–40% of all patients had hypertension. The geriatricians, however, stated that hypertension was prevalent in up to 70% of their patients and they each saw 60–100 patients per month. In Cameroon, healthcare providers reported that 40–60% of their patients had hypertension.

In Peru, healthcare providers reported varying levels of awareness related to BP control, but both awareness and control were generally low and late presentation of patients is a sign that patients do not understand the reality of their diagnosis. They mentioned that the lack of time allocated during clinical encounters with patients, due to the high volume of patients, may interfere with the healthcare providers’ ability to educate patients about home BP management. Also, the lack of coordination and communication between different primary care physicians and specialists who see the same patient, and the inability in primary care clinics to allocate resources for health record data retrieval, does not allow for an accurate diagnosis of essential hypertension. Healthcare providers expressed that this is likely a result of low education among primary care physicians, limited time available for these physicians during the patient-physician encounter, requests for referrals among patients, poor coordination, or a combination of these. Healthcare providers in Peru reported that sources of information provided by TV programs can negatively impact patients’ level of awareness. The programs that have a doctor or pharmacist as a host, presenting and discussing health problems, are sometimes not aligned with practicing doctors’ diagnoses or recommendations. Most healthcare providers from Peru reported the belief that higher levels of education in patients correlated with better awareness, attitudes towards medication adherence, and overall control.

In Cameroon, the lack of awareness was largely manifested through late presentations of patients with hypertension. Healthcare providers stated that they mostly see patients presenting with stroke or abnormal kidney function due to uncontrolled and undiagnosed hypertension. One healthcare provider from Cameroon mentioned that cultural barriers are related to a cascade of care, explaining that cultural beliefs, religious practices, and the lifestyle of Cameroonians including dietary habits may interfere with hypertension treatment regimens because they may choose to use homeopathic medicines such as “garlic pills”, vitamins, or herbs in place of prescribed medications.

### 3.3 Theme 2 from interviews: accurate measurement of blood pressure

In Peru and Cameroon, healthcare providers usually relied solely on clinical BP measurements over time to confirm and register a diagnosis of hypertension in patients’ charts. Some healthcare providers prescribed home BP monitors but reported that affordability and availability were barriers to accessing these monitors for most of their patients.

In Peru, healthcare providers reported varying methods by which they would ask their patients to perform home BP monitoring. Healthcare providers explained that they usually subjectively assess whether patients can have access to BP monitors before they prescribe home BP monitoring. Most healthcare providers explained that they ask patients to bring their BP monitors to their appointments to see if they can use the monitors and give accurate BP measurements. Geriatricians from Peru stated that they often, more than other healthcare providers, ask patients to have someone who can help them with the process, but patients were generally instructed to do it themselves. Geriatricians also noted that using the BP monitor at home may be difficult for older patients because of cognitive impairments. Like geriatricians, other healthcare providers including cardiologists who see older patients from Peru, reported that they usually ask that caregivers or partners assist patients with BP monitoring. In Peru, healthcare providers said most of their patients did not own a BP monitor and usually went to a local pharmacy to get their BP measured. Patients would then return to the doctor’s office with measurements written on a piece of paper from when they visited the pharmacist. Ambulatory BP monitoring (ABPM) was mentioned by one provider in Peru as an effective tool to get a better picture of a patient’s BP but is almost non-existent and hard to provide to patients.

In Cameroon, healthcare providers mentioned prescribing home BP monitoring to all patients. The rationale for doing so was to confirm the diagnosis of hypertension or to eliminate white coat hypertension. They said patients who did not monitor their BP complained primarily about the lack of a BP monitor. Healthcare providers reported that the cost of BP monitors was the main reason that patients do not follow through on home monitoring.

### 3.4 Theme 3 from interviews: treatment of high blood pressure

In both settings, healthcare providers explained that medication adherence, access to medications, and affordability were the main challenges to achieving optimal treatment of high BP. However, in Peru, long waiting times between appointments, especially for certain specialists such as cardiologists, cause suboptimal treatment of high BP since medications are only prescribed, re-prescribed, and purchased after seeing their doctor. The availability of medications remained a challenge to accessing treatment, as well as getting advice from non-medical experts and changing medications based on opinions from these non-experts such as drug store workers who may not have any medical or health degree.

Some healthcare providers in Peru stated that the clinical guidelines for the management of high BP included recommended lifestyle modifications, such as exercise and diet, but not home BP management. In Peru, healthcare providers also explained that patients’ self-efficacy or activation level can play a role in adherence to management plans. Some patients will change their medications on their own, stop taking them, or will take them only when they have symptoms such as headaches. Healthcare providers from Peru also stated that they try to improve medication adherence by simplifying medication regimens. Healthcare providers expressed that a simple process with few medications prescribed is likely to lead to better patient adherence to regimens. In Peru, healthcare providers referred to the Seguro Integral de Salud (SIS), which translates to “Comprehensive Health Insurance”. It offers health insurance to Peruvians who do not have private insurance and is provided for people formally employed. SIS gives priority to vulnerable populations who are in extreme poverty, which makes up 18% of the population. Increased enrollment of patients in the SIS, is a positive step towards reducing the cost of out-of-pocket expenses for medications including anti-hypertensive medications. However, some healthcare providers stated that the health policies regarding medication costs, political influences, and public health interventions do not prioritize cardiovascular disease management overall and that there needs to be a shift towards a public health approach to achieve better population-level BP control.

In Cameroon, healthcare providers mentioned the out-of-pocket costs of medications, lack of health education, and poor communication and misinformation as the main barriers to accessing and adhering to treatment. One Cameroonian healthcare provider noted that the poorly established health insurance system, limits patients' access to essential preventive cardiology care. Cameroonian healthcare providers stated that patients were not compliant with treatment. They said patients’ lack of awareness, lack of seriousness towards the management of BP, and the cost of treatment were the main barriers. Healthcare providers mentioned that lifestyle changes and modifications were usually difficult to adopt by patients as a main line of treatment. Some healthcare providers stated that adherence to medications was tied to adherence to home BP management and that a holistic approach that dealt with adherence overall would be beneficial for patients.

### 3.5 Theme 4 from interviews: patient-physician relationships

Many healthcare providers from both countries felt that the high volume of patients can negatively affect the patient-physician relationship due to insufficient time during clinical encounters. In both settings, physicians expressed that the physician-patient relationship played a major role in adherence and introducing different disease management concepts such as self-monitoring may be an added burden to the patient-physician relationship.

In Peru, some healthcare providers expressed that their relationship with their patients is framed culturally in a way that the doctor only gave information about their conditions and only advises on treatment but does not try to influence patients’ decisions. Other healthcare providers from Peru described that they felt comfortable giving patients their personal cell phone numbers to make it easier for patients to reach them when needed. Some healthcare providers mentioned that they liked to help their patients as much as they could by playing different roles on different occasions. Healthcare providers sometimes assumed the role of a social worker, a scheduler, and a psychologist to ensure that a patient's health improves. Two healthcare providers from Peru expressed distress due to constantly playing multiple roles, the lack of staff, and minimal support for physicians. Healthcare providers explained that a good patient-physician communication system should be in place before introducing home blood pressure management interventions to aid doctors and patients in communicating efficiently during working hours.

In Cameroon, respondents mentioned that patients did not always follow the advice of physicians especially when it is related to lifestyle modifications, and it was partially attributed to the lack of trust between patients and physicians. Respondents explained that it was essential for patients to first accept and understand their diagnosis before they can adhere to their treatment. Patient education would improve the physician-patient relationship over time.

### 3.6 Theme 5 from interviews: opinions about self-management and patient, systems, and provider characteristics

A broad range of key sub-themes emerged ranging from a full endorsement of self-management to strong concerns about safety, ease of self-management, and medication self-titration. Three main content analysis subthemes emerged related to patients, systems, and healthcare provider characteristics in both Cameroon and Peru. Multi-level implementation strategies including local, state, and national policies were brought up during the discussions with both Peruvian and Cameroonian healthcare providers. Concerns about systems needed to support the proposed home blood pressure management program/intervention including medication self-titration and the sustainability of the program were presented in Fig. 1.

Some providers from Peru reported feeling uncomfortable with the idea of medication self-titration and stated that patients will not be able to safely self-titrate their medications even if instructed by the doctor on how to safely do so. Some healthcare providers were also concerned that this will increase the burden on healthcare providers by having to communicate more often with patients. Also, healthcare providers from Peru were concerned that patients may see the self-titration element as taking over or diminishing their doctor’s role.

Providers from Peru were worried that patients may not be able to do this accurately and follow the instructions. Additionally, some healthcare providers expressed that the time since diagnosis may play a role in adherence to this intervention, explaining that the intervention may not work for newly diagnosed patients. Healthcare providers from Peru, especially geriatricians, felt strongly about the importance of engaging caregivers or family members in BP self-management programs. Social support was also a sub-theme in the focus group discussions from Cameroon. Geriatricians in Peru felt that patient characteristics such as age and levels of education were important factors to consider when designing self-management programs. For example, some geriatricians were concerned about polypharmacy in older patients, and the ability to handle the extra task of adjusting the number of medications.

In Cameroon, most providers showed enthusiasm about the program of home BP self-monitoring and self-titration. Respondents stated that to patients, home BP management could be cost-effective, provide home comfort when managing BP, and help to reduce the white coat effect. Healthcare providers from Cameroon also conveyed that follow-up appointments with patients will be effective since they will be more actively engaged and aware of their out-of-office measurements. For Cameroonian providers, most concerns with the intervention arose from the medication self-titration protocol. Healthcare providers raised concerns about the complexity of the protocol for patients and the difficulty in reaching a consensus between different healthcare providers about the titration protocol. Like in Peru, healthcare providers in Cameroon also listed patient-related factors such as level of education and compliance, as important determinants of program success. One of the healthcare providers suggested simplifying the protocol (i.e., using generic drug names instead of drug classes). Also, they were worried about the risk of medication overdosage and misuse of the protocol, especially for those who are not able to understand the protocol. Therefore, they emphasized the need for a clear and easy-to-follow safety plan, continuous education, training, and engagement with patients during both the introduction and implementation processes.

Cameroonian healthcare providers expressed that they would be willing to utilize the protocol with their patients because it will increase patient participation, enhance the physician-patient relationship, and facilitate the work of healthcare providers towards better BP control. Healthcare providers emphasized the importance of rigorous training of both patients and healthcare providers on how to monitor BP, use the home BP management protocol, and provide continuous feedback to either the doctor or corresponding health care staff.

## 4 Discussion

Our study is among the first to explore healthcare providers’ perspectives on BP self-management in LMICs, including home BP monitoring and medication self-titration. We used a hybrid of in-depth interviews and focus group discussions with providers who care for patients regularly from Peru and Cameroon. We found that some healthcare providers were already prescribing self-monitoring of BP to their patients but were restricted by the availability of resources to support this effort. Overall, healthcare providers were supportive of the idea of BP self-management, including self-monitoring and medication self-titrations. However, in both countries, healthcare providers had concerns about safety, sustainability, and the need for systems to support BP self-management programs.

BP self-management interventions that include medication self-titration are associated with better BP control and are cost-effective and are being increasingly utilized in high-income countries (Tucker et al., 2017, Monahan et al., 2019). To our knowledge, BP self-management interventions that include medication self-titration have not been implemented nor well-researched for feasibility and acceptability in LMICs. If proven to be feasible and acceptable by both patients and healthcare providers, self-management interventions can be a cost-effective and efficient tool in achieving better BP control in LMICs reducing friction related to compliance or tension between healthcare providers and patients (Kostova et al., 2020). Previous research has shown that patients from Cameroon and Peru were welcoming of the idea of self-management but skeptical about the ability to do self-monitoring accurately and the safety involving self-titration of medications (Al-Rousan et al., 2020). Our qualitative study offers additional evidence to support the acceptability and feasibility of self-management intervention but from the perspective of healthcare providers.

Positive healthcare provider impressions of home BP management that includes BP monitoring and medication self-titration included that it will be a cost-effective approach that provides home comfort, patient engagement, empowerment, and bridges the gaps in the patient-physician relationship. This is consistent with findings from high-income countries (Fletcher et al., 2016). Hearn et al. in a recent scoping review found self-management interventions to be an effective means of improving health outcomes (Hearn et al., 2019). This work suggests the need for patient and provider involvement in all stages of the design and development of these programs for sustainability and scalability in target settings (Hearn et al., 2019). Healthcare providers raised concerns about certain aspects of home BP management that involve the safety of patients, the readiness of local and national health systems to support such interventions, and the ability to sustain these interventions over time. Unraveling these perceived obstacles, which can challenge any future intervention, is essential as they can be addressed with anticipation in developing further interventions either through more proactive engagement with providers or by directly targeting healthcare providers, as well as patients, through multi-pronged interventions.

Healthcare providers from both Cameroon and Peru agreed that, combined with lifestyle modifications, home BP management will be a powerful tool to address the burden of hypertension among their patients. However, Cameroon’s healthcare providers seemed to show more enthusiasm about the feasibility and applicability of home BP self-management in their country than the healthcare providers in Peru. This may be explained by the fact that Peruvian healthcare providers involved were primary care physicians compared to specialists who made up the majority of healthcare providers included in this study from Peru.

A recent *meta*-analysis of non-pharmacological interventions for the prevention of hypertension in LMICs found that home-based health education reduced mean systolic BP by 2.11 mm Hg (95% CI: −3.20, −1.02 mm Hg), but they have not tested home-monitoring or medication self-titration (Mudge et al., 2015). On a patient level, providers mentioned patients’ characteristics being a barrier to home BP management. Previous research from high-income countries has shown that healthcare providers see a paradigm shift to self-management approaches from who has “control” of patient care in other diseases, as being challenging (Saif-Ur-Rahman et al., 2019, Croxson et al., 2017). However, this issue had not risen to become a separate theme in our analyses, which could be attributed to the differences in methods or culture. When coupled with health education interventions and raising awareness on hypertension among caregivers or spouses, BP control and cardiovascular health in general home BP management has the potential to engage patients in decisions related to their health and increase compliance. On a physician level, it was noted that incorporating home BP management into clinical guidelines and hospital policies would encourage physicians and specialists to prescribe home BP management and aid in its success and sustainability. A systematic review by Owolabi et al. demonstrated a dearth of hypertension guidelines in LMICs which is in line with what was systematically found to be hindering the application and support of BP self-management interventions according to healthcare providers in our study (Owolabi et al., 2016). Tucker et al. found in their systematic review that home monitoring alone is insufficient to drive a clinically significant reduction of BP (Tucker et al., 2017). Therefore, co-interventions such as self-management are needed to reduce BP.

Affordability remained a barrier to patients purchasing monitors or affording different medications. Healthcare providers suggested using political influences to prioritize chronic disease management and cardiovascular health when financing health systems. A strong patient-physician relationship can facilitate successful home BP management in patients. Additionally, it is important to create infrastructure and team-based care that can support physicians in their efforts if they are expected to educate patients on self-titration and monitor patient safety as they self-titrate medications.

To increase adherence and reduce the burden of home BP management and self-titration to patients with comorbidities or who are older in age, several approaches have been suggested. First, on a system level, BP self-management should be incorporated within clinical and hospital guidelines. Second, coordination with local pharmacies could increase the availability of medications, BP monitors, and improve guidance aligned with doctor’s orders. Third, reduction of the cost of medications might be achieved using a polypill-based approach.

Although this study fills an important gap in the literature on healthcare providers’ perspectives and attitudes towards home BP management in LMICs, our study has several limitations. First, the study was limited in that healthcare providers were all recruited from the same hospital in each country, which may limit generalizability, despite maximal variability sampling. However, hospitals were selected because they attract a variety of patients from different geographic locations throughout Peru and Cameroon but since there may be health and economic inequalities within most LMICs our results may not be generalizable to both countries. Second, we used grounded theory which provides a systematic and rigorous approach for rich data analysis that can be used to develop data-driven theories. However, data analysis and interpretation are subject to researcher-induced bias (Fassinger, 2005). By conducting focus groups and in-depth interviews with physicians, specialists, and nurses in Peru and Cameroon, a wide variety of practices and experiences in managing patient use of BP monitors at home has been elicited. The inclusion of nurses in the focus groups in Cameroon may have shed more light on how self-monitoring fits within the wider structure of primary care than interviewing physicians alone. Third, the method of data collection in each setting was dictated by practical issues such as obtaining internal healthcare facility approvals and the availability of practitioners. Data collectors in both settings had different training backgrounds which may explain the differences in probing. This also may have been reflected in the feasibility and acceptability subthemes where the Cameroon healthcare providers seemed to be generally more supportive of self-management interventions compared to the Peru healthcare providers. The thematic analysis was limited in that it was principally completed by two researchers (TA and AD); however, the analysis of one of the transcripts by three researchers, and the subsequent discussion of themes arising, ensured that themes were robust. Lastly, self-management practices vary according to being diagnosed with other comorbidities and that may explain Peru’s healthcare providers' (cardiologists and geriatricians) concerns about safety and sustainability. It would have been ideal to compare themes from specialists, then to those from primary care healthcare providers in the same country, but this limitation has been mitigated by interviewing both types of healthcare providers in two LMICs using the same semi-structured guide.

## 5 Conclusions

According to healthcare providers in Peru and Cameroon, home BP management that includes medication self-titration may be a feasible intervention with healthcare providers although concerns were noted. Factors that healthcare providers believe can improve home blood pressure management interventions include healthcare provider engagement, encouraging patient education, simplifying self-titration algorithms and protocols, family support, reducing the costs of BP monitors and medications, and ensuring policies and clinical guidelines support home BP management and team-based care. By providing support and resources to healthcare providers, it can facilitate successful home BP management interventions. Local contexts should be taken into consideration when designing home BP management interventions and healthcare providers' perspectives can help shape these interventions.

**Funding**: Dr. Al-Rousan is supported by a grant from the 10.13039/100000050National Heart, Lung, and Blood Institute (K23HL148530).

## CRediT authorship contribution statement

**Tala Al-Rousan:** Conceptualization, Data curation, Formal analysis, Software, Writing – original draft, Writing – review & editing. **Mina Awad:** Data curation, Formal analysis, Software, Writing – review & editing. **M. Amalia Pesantes:** Data curation, Formal analysis, Software, Writing – review & editing. **Namratha R. Kandula:** Methodology, Resources, Validation, Writing – review & editing. **Mark D. Huffman:** Methodology, Resources, Validation, Writing – review & editing. **J. Jaime Miranda:** Methodology, Resources, Validation, Writing – review & editing. **Rafael Vidal-Perez:** Methodology, Resources, Validation, Writing – review & editing. **Anastase Dzudie:** Data curation, Methodology, Resources, Validation, Writing – review & editing. **Cheryl A.M. Anderson:** Conceptualization, Methodology, Resources, Validation, Writing – review & editing.

## Declaration of Competing Interest

The authors declare the following financial interests/personal relationships which may be considered as potential competing interests: In the last 3 years, Mark D Huffman has received support from the World Heart Federation through unrestricted education grants from Boehringer Ingelheim, Novartis, and Bupa and from the American Heart Association, Verily, and AstraZeneca for work unrelated to this research. Mark D. Huffman has also received salary support from the American Medical Association for his role as an associate editor for JAMA Cardiology and has planned patents for combination therapy for the treatment of heart failure. The George Institute for Global Health where Mark D. Huffman works has a patent, license, and has received investment funding with the intent to commercialize fixed-dose combination therapy through its social enterprise business, George Medicines.

## Data Availability

Data will be made available on request.
